# Physiological and Proteomics Analyses Reveal Low-Phosphorus Stress Affected the Regulation of Photosynthesis in Soybean

**DOI:** 10.3390/ijms19061688

**Published:** 2018-06-06

**Authors:** Shanshan Chu, Hongyan Li, Xiangqian Zhang, Kaiye Yu, Maoni Chao, Suoyi Han, Dan Zhang

**Affiliations:** 1 Collaborative Innovation Center of Henan Grain Crops, College of Agronomy, Henan Agricultural University, Zhengzhou 450002, China; chushan3@163.com (S.C.); 15038043148@163.com (H.L.); 15333717403@163.com (X.Z.); 17638110601@163.com (K.Y.); 2 Henan Institute of Science and Technology, Henan Collaborative Innovation Center of Modern Biological Breeding, Xinxiang 453003, China; chaomaoni@126.com; 3 Industrial Crops Research Institute, Henan Academy of Agricultural Sciences, Zhengzhou 450002, China

**Keywords:** low-phosphorus stress, photosynthesis, proteomics, leaf structure, gene expression

## Abstract

Previous studies have revealed a significant genetic relationship between phosphorus (P)-efficiency and photosynthesis-related traits in soybean. In this study, we used proteome profiling in combination with expression analysis, biochemical investigations, and leaf ultrastructural analysis to identify the underlying physiological and molecular responses. The expression analysis and ultrastructural analysis showed that the photosynthesis key genes were decreased at transcript levels and the leaf mesophyll and chloroplast were severely damaged after low-P stress. Approximately 55 protein spots showed changes under low-P condition by mass spectrometry, of which 17 were involved in various photosynthetic processes. Further analysis revealed the depression of photosynthesis caused by low-P stress mainly involves the regulation of leaf structure, adenosine triphosphate (ATP) synthesis, absorption and transportation of CO_2_, photosynthetic electron transport, production of assimilatory power, and levels of enzymes related to the Calvin cycle. In summary, our findings indicated that the existence of a stringent relationship between P supply and the genomic control of photosynthesis in soybean. As an important strategy to protect soybean photosynthesis, P could maintain the stability of cell structure, up-regulate the enzymes’ activities, recover the process of photosystem II (PSII), and induce the expression of low-P responsive genes and proteins.

## 1. Introduction

Crop growth and yield formation rely on photosynthesis, but the photosynthetic process largely relies on phosphorus (P) and P-containing compounds [1,2]. P is the composition of chloroplast and photosynthesis and affects the structure and function of photosynthetic organs [3]. Thus, P plays a key role in photosynthesis, and photosynthesis is sensitive to low-P stress. The increase of crop yield requires improved photosynthesis [4]. Thus, efficient use of P is a potentially important determinant of plant photosynthesis, further benefiting crop growth and yield.

P is an essential macronutrient for plant growth and development, but the P resources used for producing fertilizers is presumed to be used up in the future [5]. As an important crop grown worldwide providing both protein meal and vegetable oil for consumption, soybean accounts for more than 50% of the global oilseed production [6]. However, soybean is also a high P demand species, and low-P stress is a key factor constraining soybean growth and yield [7]. Compared with other nutrient deficiencies and toxicities, P deficiency has been one of the largest limits affecting soybean production [8]. It has been reported that more than 5.7 billion ha of land worldwide is deficient in plant-available P [9,10], consequently resulting in the reduction of average global soybean production.

Our previous genetic studies have demonstrated that there is a significant positive correlation between P efficiency and photosynthetic-related traits in soybean, particularly under low-P stress [2]. We have also observed that leaves differed greatly in morphology and structure under low-P conditions, with soybean leaves grown under P deficiency showing a smaller size compared with those fertilized with sufficient P [11], further suggesting that P deficiency may decrease photosynthesis and ultimately affect soybean growth and yield. These findings were also observed in other studies showing that the soybean leaves developed under low-P stress are smaller and thicker, with fewer chloroplasts and thinner grana stacks compared with those in sufficient P-fertilized leaves [3,12,13]. Moreover, some other physiological and biochemical changes in responses to low-P stress were also observed. For examples, during low-P stress, leaves have lower levels of chlorophyll content, gas exchange, and the activity of enzymes related to photosynthesis than sufficient P-fertilized leaves [14,15]. P status in plant tissue directly influences photosynthetic metabolic processes of energy transfer (ATP, nicotinamide adenine dinucleotide phosphate, NADPH), regeneration of substrates and utilization of photosynthates, and CO_2_ diffusion inside the soybean leaves [16,17]. Accordingly, plant leaves have a lower photosynthetic capacity per leaf area when experiencing low-P stress, resulting in the inhibition of plant growth and yield decrease. Although great progress was made, the physiological and molecular changes of soybean photosynthesis in responses to low-P stress remains to be further elucidated.

Recently, we performed a comparative transcriptome analysis in soybean leaves under low-P stress, finding that the soybean responses to low-P stress is a complicated process involving several metabolic reactions, such as photosynthesis, nitrogen metabolism, carbohydrate metabolism, flavonoid biosynthesis, energy metabolism, signal transduction, and protein synthesis [11]. Specifically, genes involved in light harvesting, energy transfer, light energy conversion, ATP synthesis, and CO_2_ fixation were significantly differentially expressed, suggesting that photosynthesis and the related metabolic processes might be significantly affected by low-P stress. In this study, we expand our previous studies [11] to characterize the expression patterns of several importantly photosynthesis-related genes, such as rubisco activase (RCA), to further elucidate the possible mechanism of how low-P affects photosynthesis in soybean. RCA has a demonstrated role in catalyzing the activation of rubisco, which is the key photosynthesis enzyme [18], which is one of the several new targets potentially involved in improving plant yield [19]. The single-nucleotide polymorphisms (SNP) in RCA (*GmRCAβ*) promoter have been detected, and have shown a strong positive correlation with its expression level diversity in soybean [18]. Considering the gene expression of *GmRCAβ* was affected by either or both low-P stress and polymorphism in the promoter, we here attempted to explore if the *GmRCAβ* expression level and the genetic polymorphisms are associated with soybean P efficiency.

On the other hand, taking into account that transcriptomic profiling sometimes fails to unequivocally reveal regulations of biological processes, for example, due to gene redundancy or post-translational modifications [20,21], in this study, a proteomic approach combined with induced-expression analysis, leaf ultrastructural analysis, and biochemical investigations were applied to further elucidate the regulatory mechanism of soybean photosynthesis in response to low-P stress. The goals of this study were (i) to investigate the physiological and molecular regulatory mechanism of soybean photosynthesis in response to low-P stress; and (ii) to provide useful information for further research regarding the selection of the candidate genes involved in photosynthesis and P stress responses.

## 2. Results

### 2.1. Low-P Stress Affected Soybean Plant Growth and Leaf Structure

**Figure 1:**
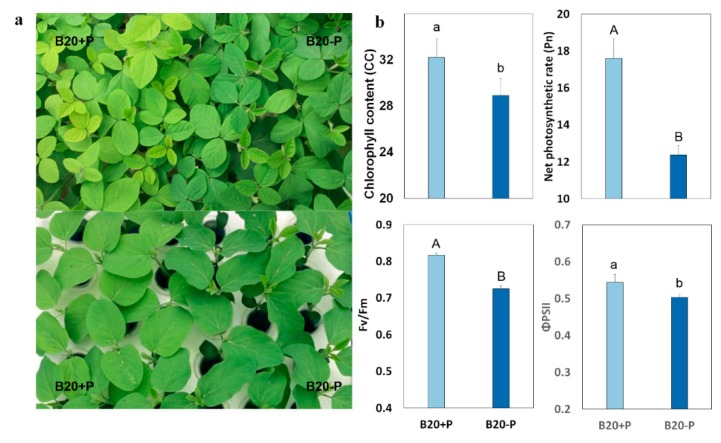
Effects of low-P stress on morphology and photosynthetic parameters of soybean seedling. Five days old germinated plants were transferred on Hogland nutrient solution (Optimum P, +P, 1000 μmol KH_2_PO_4_; Low-P, −P, 5 μmol KH_2_PO_4_) and allowed to grow for two weeks under hydroponic conditions. (**a**) Soybean grown under optimum P conditions (left) and  −P conditions (right); (**b**) The effects of low-P stress on soybean photosynthetic parameters chlorophyll content (CC), net photosynthetic rate (Pn), maximum quantum efficiency of PSII (Fv/Fm) and quantum efficiency of PSII (ΦPSII). ANOVA was used to compare significance and the lowercase/capital letter denote significantly different at *p* < 0.05/0.01 (Least Significant Difference, LSD test).

After a two-week treatment, soybean grown under low-P stress had a lower shoot dry weight (SDW), total dry weight (TDW), P concentration (PC), and P uptake (PUP), while the root dry weight (RDW), root:shoot weight ratio, P use efficiency (PUE), and acid phosphatase activity (APA) were higher compared with those of soybean plants grown at optimum P conditions (Table 1). Compared with light green leaves of the plants grown under optimum P condition, the leaf color of the plants grown under low-P stress turned dark green and the leaf area was smaller (Figure 1).

**Figure 2:**
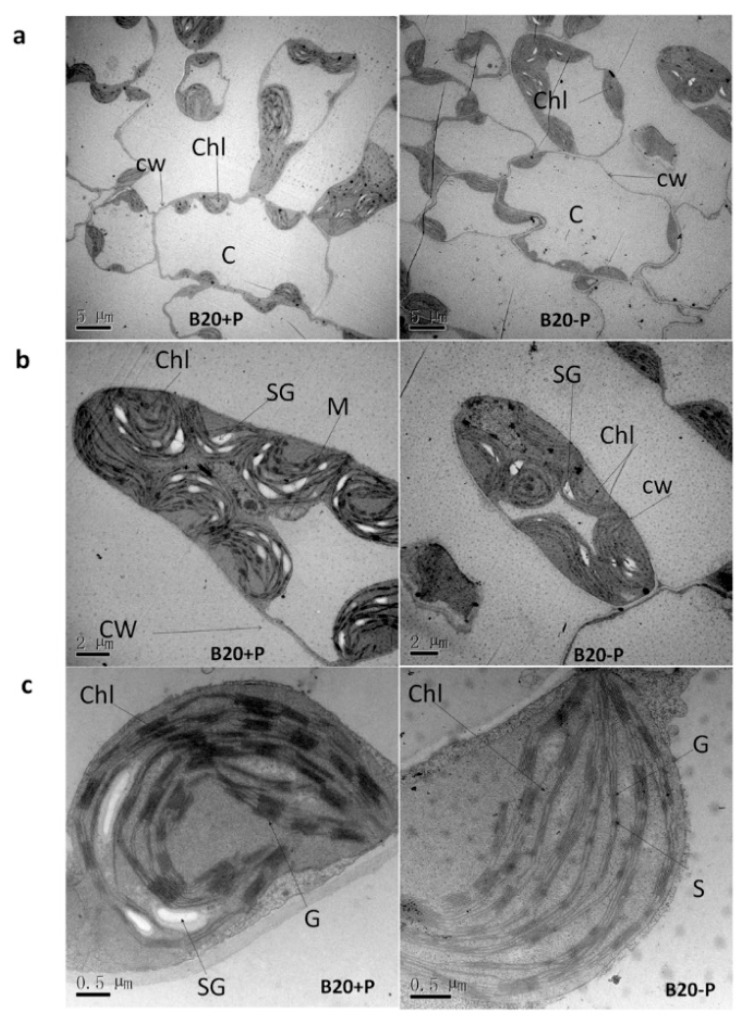
Electron micrographs of leaf mesophyll of soybean grow under low-P condition (−P, 5 μmol KH_2_PO_4_) and control (optimum P, 1000 μmol KH_2_PO_4_). (**a**–**c**) Changes in organelles at different scales in 5, 2, and 0.5 μm, respectively. Chl: chloroplast; C; cell, CW: cell wall; SG: starch granule; M: mitochondrion; G: grana; S: stenotrophomonas granule.

To further analyze if P deficiency affects photosynthetic organs, we investigated leaf structure under a transmission electron microscope (Figure 2). After low-P treatment, we observed that the junctions between cells had become extremely loose and the cell separation had increased considerably resulting in very large intercellular spaces (Figure 2a). This observation suggests that a decrease in the barrier resistance might occur after low-P stress. In addition, the ultrastructure of the chloroplasts was also affected after low-P treatment. For example, the external form of chloroplasts in plants grown under low-P stress conditions were oval instead of circular in optimum P-fertilized leaves, the number of chloroplasts and grana significantly decreased (Figure 2b), the chloroplasts swelled and disorder of the thylakoid arrangement were observed, and grana gradually became hypogenetic and eventually dissolved. For example, a significant increase in stroma thylakoids can be observed in low-P plants compared with control (Figure 2c). Therefore, these abnormal morphological and anatomical structure observed in low-P affected plants suggest that P plays important roles in the development of chloroplast components and sufficient P supply is critical for the successful implementation of photosynthesis in soybean.

### 2.2. Low-P Stress Affected Photosynthetic Functions

To further determine if photosynthesis was also affected by low-P stress, we also determined chlorophyll content (CC), chlorophyll fluorescence, and gas exchange parameters. We found that P deficiency significantly decreased the net photosynthetic rate (Pn), stomatal conductance (Gs), transpiration rate (Tr), the maximum quantum efficiency of PSII (Fv/Fm), and the quantum efficiency of PSII (ΦPSII), but increased the intercellular CO_2_ concentration (Ci), suggesting that the decreased photosynthetic rate might be controlled by non-stomatal limitation (Table 1 and Figure 1b). Meanwhile, low-P resulted in the reductions in the initial activities of ribulose-1,5-bisphosphate (RuBP) less (by 18.8%) and ATP synthetase (by 13.6%). These results indicate that photosynthetic CO_2_-fixation in low-P stressed plants may be limited by RuBP regeneration and ATP and/or NADPH supply (Table 1).

### 2.3. Low-P Stress Affected the Expression of the Genes Involved in Photosynthesis

Our previous RNA-Seq studies have shown that many genes involved in photosynthesis showed a significant change in the expression during low-P treatment in B20 [22]. These differentially-expressed genes (DEGs) were found to be mainly involved in photosynthesis, Calvin cycle, light reaction, carbon metabolism, photoreceptor, and photoprotection. Here, to further assess if the low-P stress affected the gene regulatory levels of photosynthesis, we randomly selected 14 of the photosynthetic-related DEGs and examined their expression pattern after low-P treatment using quantitative real-time PCR (qPCR).

**Figure 3:**
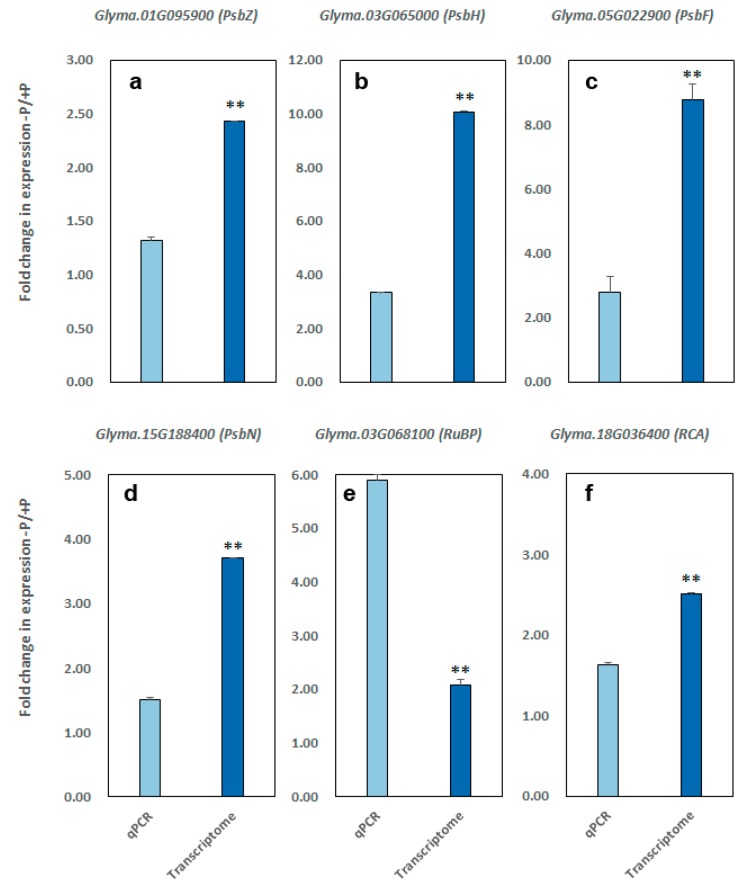
Relative transcript levels by transcriptome and *q*PCR of photosynthetic related genes under low-P condition (−P, 5 μmol KH_2_PO_4_) and control (optimum P, 1000 μmol KH_2_PO_4_). (**a**–**d**) *Glyma.01G095900, Glyma.03G065000*, *Glyma.05G022900*, and *Glyma.15G188400* encode photosystem II PsbZ protein, photosystem II PsbH protein, photosystem I subunit III PsaF protein, and photosystem II PsbN protein. (**e**–**f**) *Glyma.03G068100* and *Glyma.18G036400* encode ribulose-1,5-bisphosphate carboxylase and rubisco activase. Data are means  ±  SD of three replicates. Student’s *t*-test was used to compare the significance. * indicates significant differences at *p* < 0.05, ** indicate *p* < 0.01.

In the result, a good consistency in the expression pattern of the selected gene was observed between the RNA-seq result and qPCR assay (Table 2 and Figure 3), with 11 of the genes showing significantly down-regulated expression after the treatment. For example, four genes, PsbZ (*Glyma.01G095900*), psbN (*Glyma.15G188400*), PsbH (*Glyma.03G065000*), and PsbK (*Glyma.15G248600*), that encode the components of the PSII complex, showed 1.3–3.3-fold downregulation in the expression. Similarly, ATP synthesis coupled proton transport (*Glyma.12G096200*), which is involved in photosynthetic electron transport, ribulose-1,5-bisphosphate carboxylase (*Glyma.03g068100*), pyruvate orthophosphate dikinase (*Glyma.17G020600*), fructose-biphosphate aldolase (*Glyma.20G122500*), Rubisco activase (*Glyma.18G036400*) and phosphoenolpyruvate carboxylase (*Glyma.06G277500*), as well as the photosystem I subunit III protein (PsaF, *Glyma.05G022900*), were downregulated. In this study, the value of Fv/Fm significantly decreased in the leaves under low-P stress for 14 days (Figure 1), indicating that P deficiency had an adverse effect on PSII in soybean. This observation is consistent with the findings in which the decrease in the value of Fv/Fm were also found when limited P were applied to other plant species, such as marine diatom [23] and maize [3]. On the other hand, we also found that several genes were significantly up-regulated, such as an NADP^+^-dependent malic enzyme gene (*Glyma.08G201200*) and two pyruvate kinase genes (*Glyma.13G149800*, *Glyma.19G190100*) (Table 2 and Figure 3). These results suggest that low-P stress has strongly affected various regulatory pathways involved in the photosynthesis in soybean.

### 2.4. Photosynthesis-Related Proteins Were Differentially Expressed after Low-P Stress

**Figure 4:**
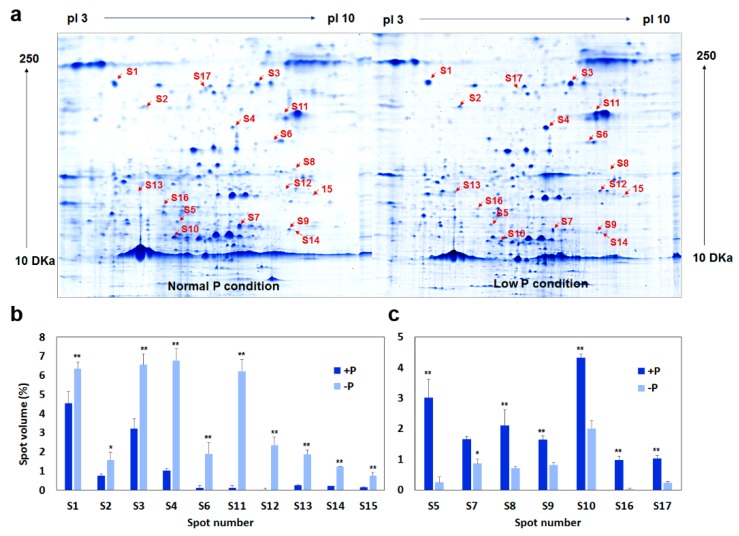
Representative CBB-stained 2D-IEF/SDS-PAGE gels of proteins extracted from soybean leaves grew at low-P condition (−P, 5 μmol KH_2_PO_4_) and control (optimum P, 1000 μmol KH_2_PO_4_) (**a**) and the relative spot volumes of the 17 significantly increased (**b**) and decreased (**c**) protein spots in low-P stress and control leaves of soybean. Bars represent means ± SD. Student’s *t*-test was used to compare significance. * and ** denote significant low-P treatment differences at *p* < 0.05 and 0.01, respectively.

Our results above have shown that low-P stress has caused significant changes in physiological traits and the levels of the transcriptome, whereas the change in the level of proteomics remains unclear. To gain a comprehensive understanding of how low-P stress affects the expression of the proteome, we comparatively examined the expression profiling of the proteome of B20 grown on optimum P and low-P conditions. The two-dimensional gel analysis identified a total of 55 spots that showed significant differences in protein relative abundance (*p* < 0.05) between the two conditions. These proteins were temporally designated as low-P induced differentially-expressed proteins (DEPs). Of the 55 DEPs, 33 could be identified and annotated using peptide mass fingerprinting (PMF). Based on the functional descriptions and related literatures, we found that 17 of 33 DEPs were involved in several important photosynthesis-related processes (Table 3, Tables S1 and S2), such as carbon metabolism, photoreceptor proteins, light reaction, and photoprotection, suggesting that low-P stress also affected the regulation of photosynthetic mechanism at the protein level other than the levels of gene regulation and physiological and transcribed. Figure 4 shows the expression patterns of 17 DEPs on two representative gel images under optimum P and low-P conditions, respectively.

The 17 DEPs involved in photosynthesis showed different expression patterns under optimum P and low-P conditions. Ten of the 17 DEPs shown were significantly induced, while the remaining seven showed lower abundance in low-P treated leaves compared those in control leaves (Figure 4 and Table 3). The down-regulated proteins include a triosephosphate isomerase (S5), an ATP synthase (S7), a rubisco activase (S8), an iron-sulfur protein (S9), a phytochrome A (S10), a peroxisomal NAD-malate dehydrogenase (S16), and a cryptochrome 1 (S17) (Table 3 and Figure 4). This finding is consistent with our previous results where the enzyme activities of ATP synthase and rubisco activase were significantly decreased at both levels of physiological and biochemical experiments (Table 1).

It is worth noting that an RCA gene, *Glyma.18G036400*, which was detected in both transcriptomic and proteomic (S8) analyses, and its expression were significantly decreased under low-P conditions, suggesting the pivotal importance of this RCA gene in the response and adaptation of leaves to low-P stress. In addition, the roles of ATP synthase and the cytochrome in limiting chloroplast electron transport and determining photosynthetic capacity [24]. In this study, the chlorophyll content and fluorescence parameters in leaves under low-P stress were lower than those in controls grown at optimum P conditions (Table 3), which may be relevant to the significant decrease in the expressions of these photosynthetic-related enzymes. The proteomics and physiological data presented here showed that soybean leaves have a lower photosynthetic capacity due to its lower photosynthetic rate, chlorophyll fluorescence parameters, and the lower expression levels and activities of Calvin cycle enzymes during low-P stress.

### 2.5. Association Analysis for the SNPs of GmRCAβ Promoter

Rubisco activase (RCA), which catalyzes the activation of the key photosynthesis enzyme Rubisco [18], has been verified to be a new target potentially involved in plant yield improvement [19]. Moreover, the variation in rubisco activase (*GmRCAβ*) gene promoters were associated with its expression level in soybean [18]. In our present study, consistently, we found that low-P stress also affects the expression of *GmRCAβ* gene. Considering the *GmRCAβ* gene expression was affected by both low-P stress and polymorphisms of the promoter, therefore, it is reasonable to presume that the genetic polymorphisms of *GmRCAβ* and its expression might associate with soybean P efficiency.

The polymorphism data of *GmRCAβ* promoter was provided and described [18]. In brief, a 2300-bp region containing the promoter and the 5′-UTR region was sequenced in a natural population comprising 219 soybean accessions originated from different geographic regions in China. Sequence alignment showed 16 SNPs and five haplotypes, which were classified as two groups (group A: types 1, 2, and 3; group B: types 4 and 5) [18]. Moreover, the expression level of the genes was significantly higher with promoters for group A compared with group B, suggesting a correlation between polymorphisms/haplotypes in the promoter region and the expression level of *GmRCAβ*.

**Figure 5:**
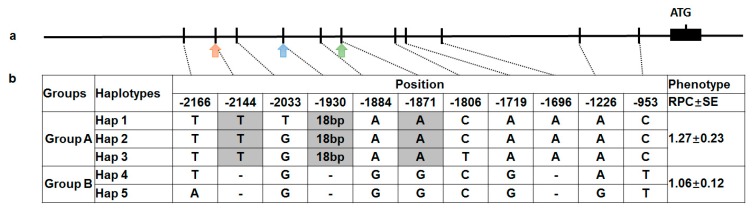
Candidate gene association analysis of the *GmRCAβ* promoter region and relative P concentration in 219 soybean accessions. (**a**) The *GmRCAβ* gene model showing the allelic variation (frequency > 5%) of the *GmRCAβ* promoter sequence. Orange arrows indicate the TATA boxes, blue arrow indicates the AT1 motif, and green arrow indicates the ARE; (**b**) Nucleotide polymorphisms in the promoter region of *GmRCAβ*, and two groups (Group A: Hap1, 2 and 3; Group B: Hap 4 and 5) are listed. Significant polymorphic functional nucleotide sites are indicated in gray. The haplotype effects were estimated based on the mean relative P concentration (RPC). RPC: relative P concentration under optimum P and −P.

In this study, to verify if the polymorphisms/haplotypes in the *GmRCAβ* promoter region were the causal sites for soybean P efficiency [7], candidate gene-based association analysis was performed by mixed linear model (MLM) analysis using TASSEL 5.0 software [25]. After association analysis, nine SNPs significantly associated with the P efficiency were identified (Figure 5). Interestingly, three of these significant SNPs were located within three regulatory elements, respectively. For example, the TATA box, the anaerobic induction-responsive element, and the AT-1 motif. In addition, to determine if the nine putative causative sites have joint effects affecting P efficiency, we analyzed the associations between the haplotypes and P-efficiency-related phenotypes. As a result, the relative phosphorus concentration (RPC) values were highest in types 1–3, which combined with the favorable alleles. Overall, the five haplotypes can explain 35% of the phenotypic variation (*p* < 0.01, ANOVA) for the RPC, and a 1.2-fold difference was observed between two groups.

## 3. Discussion

Plant growth and function are intimately linked to the acquisition of Pi and the functioning of the photosynthetic machinery [26]. Moreover, P is essential for the maintenance of light reaction, photophosphorylation, and the production of carbohydrates, however, it is also one of the most difficult macronutrients for plants to obtain. Soybean, an important crop sensitive to P deficiency, has been demonstrated that there is a significant correlation between P efficiency and photosynthetic capacity [2]. In this study, our results illustrate that a stringent relationship between P supply and the genomic control of photosynthesis exists in soybean. Low-P stress affected soybean photosynthetic capacity as reflected in the striking decline of leaf photosynthetic, plant shoot biomass, and shoot:root weight ratio (Table 1). This was underpinned by alterations in the leaf morphological and anatomical structure, enzyme activity and the expression of genes and proteins related to the photosynthetic process.

Leaf anatomical structure plays crucial roles in the regulation of photosynthetic capacity, providing a structural framework for the gases diffusion and the photosynthetic function optimization [26,27]. Generally, a higher photosynthetic rate benefits from more robust chloroplasts and grana and a much faster metabolite transfer between the mesophyll cells [28]. Accordingly, low-P stress decreased the chloroplasts, grana number, leaf area, and increased intercellular spaces. All these anatomical features in leaves (Figure 1 and Figure 2), might be partially responsible for the depressed photosynthetic capacity.

In addition, reduced photosynthetic capacity not only affected the morphological and anatomical structure, but also the physiological and biochemical processes, which was observed in the changes in the expressions of the functional genes and proteins (Table 3). In this study, the abundance of several proteins associated with the key enzymes of Calvin cycle, such as ribulose-1,5-bisphosphate carboxylase (rubisco), rubisco activase (RCA), phosphoenolpyruvate carboxylase, ATP synthase, and malate dehydrogenase were all downregulated in the plants grown under low-P conditions (Table 3). It is widely recognized that rubisco catalyzed RuBP reacts with CO_2_ to produce PGA. Then PGAld is formed in the presence of NADPH and ATP, and PGAld is transformed into RuBP in Calvin cycle [29]. Our results shown that the RuBP in leaves may slow down under low-P stress. The decline in the photosynthetic rate could, therefore, be partially attributed to the depression of RuBP regeneration under low-P stress. RCA is generally regarded as a crucial enzyme in photosynthesis [30,31]. Recently, Yin has identified that a soybean RCA (*GmRCAβ*) not only affects the photosynthetic rate but also significantly correlates with plant productivity and seed yield [19]. In this study, a decrease in the photosynthetic rate with down regulation of RCA was consistently observed under low-P conditions (Table 3). Moreover, candidate gene-based association analysis revealed a significant association between P concentration and the polymorphisms of the *GmRCAβ* promoter which determines the gene expression level. Accordingly, we concluded that both rubisco activity and RuBP regeneration capacity are affected by P deficiency. This is largely due to the decreases in the amount of rubisco, RCA, and the activation of these enzymes under P deficiency [16].

Research reports that low-P stress might be damage PSII process and decrease Fv/Fm [15,32], which has been widely used to represent the maximum potential quantum efficiency of the PSII process. In the present study, the Fv/Fm value significantly decreased under low-P condition for 14 days (Figure 3A), indicating that low-P stress had a severe effect on PSII in soybean. This decrease is inconsistent with the observations of P limitation in some marine pico-phytoplankton [15,33], and this difference may be due to the duration of P stress. It has been reported that most significantly over-represented genes during early P deficiency encoded proteins related to the photosynthetic light reactions and that the expression levels of these genes continuously declined during late P depletion [23]. Furthermore, our results also showed that many genes and proteins involved in the light reactions in photosystem II (PSII) and photosystem I (PSI) were significantly downregulated under low-P stress (Table 2 and Table 3), suggesting a close relationship between P supply and the genomic control of photosynthesis. Downregulation of the genes/proteins may further reduce the photosynthetic machinery, finally beneficial to a cell being subjected to a nutritive imbalance by reducing the generation site of reactive oxygen species.

Although the leaves’ color becomes darker, observed to be due to P deficiency, the chlorophyll content did not significantly increase, and even the proteins related to photosynthetic pigments were clearly downregulated for several folds in abundance (Table 3). Thus, we might conclude that the darker color of the leaves may be a result of the obstruction of the transport of triose phosphate which was caused by the decreases in P concentration and the abundance of triosephosphate isomerase in leaves (Table 3). Moreover, rubisco has been also proven to inhibition by unproductive binding of sugar-phosphates that lock active sites in a closed conformation [34], ultimately reducing photosynthetic capacity. Additionally, P deficiency also caused the decrease in the expressions of ATP synthase and iron sulfur proteins (Table 3), which may limit the production of ATP and NADPH to a certain extent. Thus, the low-P stress caused a decline of the electron transport rate and formation of assimilatory power, indicating that these two aspects may be important regulatory factors for photosynthesis under low-P conditions. 

On the other hand, although low-P stress has a great influence on soybean photosynthesis, there were still some genes and proteins found to be upregulated, such as pyruvate kinase and UDP glucose 6-dehydrogenase. These upregulated genes and proteins may be involved in accelerating ATP synthesis through the TCA cycle as a photoprotective mechanism. This result is consistent with observations of P limitation in maize, which caused the upregulation of some proteins [3]. In soybean, although extensive studies have been made related to the process of photosynthesis, most previous studies have been mainly focused on a few regulatory steps of light acclimation and did not reveal a potential regulatory network. In the present study, under low-P conditions, the decline of photosynthesis in soybean was found to be involved the regulation of enzymes involved in the Calvin cycle, absorption and transportation of CO_2,_ leaf structure, and assimilatory power formation.

In conclusion, our results showed that low-P stress can inhibit photosynthesis capacity and growth of soybean plants by inhibiting ATP synthesis and carbon assimilation, damaging the structure of PSII, and impairing the leaf anatomical structure. Our results shed light on the mechanisms involved in the response to low-P stress in soybean and indicated the involvement of P efficiency and supply in photosynthesis at the levels of physiology, phenotype, gene, and protein relative abundance. Future studies will be conducted to clarify the molecular mechanism of how P ameliorates the effects on soybean photosynthesis.

## 4. Materials and Methods

### 4.1. Plant Materials and Hydroponics Experiments

In our previous study, a soybean transgressive recombinant inbred line, B20 (low-P tolerant genotype), was used for transcriptomics analysis under two different P levels, low-P, and optimum P supply [11,22]. We have observed that leaves of B20 differ greatly in morphology and structure (such as smaller leaves) under low-P conditions. Moreover, we also found that the expression of many genes involved in photosynthesis was significantly affected in B20 after low-P stress. In this study, the genotype B20 was used to characterize physiological and molecular responses to low-P stress using proteome profiling, expression analysis of selected genes, biochemical investigations, and leaf ultrastructural analysis.

Hydroponic experiments were carried out in an artificial climate chamber under controlled conditions (day/night temperature of 28/20 °C and a 10 h light/14 h dark photoperiod) as previously described [11]. Briefly, the soybean seeds were surface-sterilized with chlorine for no more than 4 min and rinsed with running tap water until chlorine is completely removed and germinated in sterile vermiculite. Seedlings were grown in a 60-hole hydroponic tank (70 × 50 × 30 cm) with one plant per hole. Five-day old plants were transferred into modified one-half Hoagland’s nutrient solution (optimum P, 500 μmol KH_2_PO_4_; −P, 5 μmol KH_2_PO_4_), and the plants grown under hydroponic conditions for two weeks were used for proteomic profiling, gene expression analysis, biochemical investigations, and leaf ultrastructural analysis. The soybean plants were placed in the hydroponics box using a completely randomized block design, and five experimental replicates (pH = 5.8 and the solution was renewed every three days) were used. Soybean fully-developed leaves were harvested for protein and RNA extractions, respectively, and stored in a −80 °C freezer for further use.

### 4.2. Measurement of P Efficiency and Photosynthesis-Related Traits

A total of 19 P efficiency- and photosynthetic-related traits of B20 plants grown under low-P- and optimum P conditions were investigated. These traits include root dry weight, shoot dry weight, total plant dry weight, root/shoot weight ratio, P concentration, P uptake, P use efficiency, acid phosphatase activity (APA), chlorophyll content, net photosynthetic rate, stomatal conductance, transpiration rate, intercellular CO_2_ concentration, maximum quantum efficiency of PSII, quantum efficiency of PSII, photochemical quenching, non-photochemical quenching, ribulose-1,5-bisphosphate carboxylase, and ATP synthase activity.

Plant enzymes were deactivated by heating-induced denaturation at 105 °C for 60 min; the samples were then oven-dried at 65 °C for three days. The dried samples were milled and subsequently digested with concentrated H_2_SO_4_ and H_2_O_2_ to facilitate the determination of P concentration using the molybdate-blue colorimetric method [35]. P-efficiency in the plant (P-use efficiency) was defined as the mg of plant dry weight produced per mg of P absorbed by plants, while P absorption efficiency was defined as total P in the plant (mg plant^−1^).

Soybean leaf samples (approximately 0.1 g) were collected from each of five plants (biological replicates). Leaf samples were fine powdered with liquid nitrogen and macerated in 1 mL of extraction buffer (50 mM sodium acetate buffer, pH 4.0). Then, the extract was centrifuged at 16,000× *g* at 4 °C for 10 min. The 490 µL of extraction buffer was mixed with supernatant (10 µL) for APA assay with ρ-nitrophenol phosphate (ρ-NPP) as the substrate [36]. The reaction of APA was performed at 37 °C for 10 min and was terminated using 1 mL of 1 M NaOH, and the OD value was measured at 410 nm in a spectrophotometer. Rubisco and ATP synthase activity in soybean leaves were assayed using commercially-available plant ribulose-1,5-bisphosphate carboxylase/oxygenase (rubisco) and plant ATP synthase ELISA Kit (CUSABIO, Wuhan, China), respectively, following the manufacture’s protocols.

Photosynthesis-related traits were determined following the methods as previously described [37]. Briefly, photosynthesis-related traits including photosynthetic rate (PN), intercellular carbon dioxide concentration (Ci), transpiration rate (Tr), and chlorophyll a fluorescence were measured using a LI-6400 portable photosynthesis system (Li-Cor Inc., Lincoln, NE, USA) and PAM fluorimeter (PAM2100, Heinz Walz, Effeltrich, Germany), respectively. Three of five replicates were used for the measurement. Leaf material was dark-adapted for 20 min prior to the measurement of chlorophyll fluorescence using a leaf chip.

### 4.3. Determination of Gene Expression by qPCR

Quantitative real-time PCR (qPCR) assays were used to determine the expression level of gene (three biological and technical repeats) by an ABI 7500 system (Applied Biosystems, Foster City, CA, USA), The PCR reaction contained 0.5 μL of 10 μmol L^−1^ primers, 50 ng cDNA, and 10 μL of real-time PCR SYBR MIX (QPK-201; TOYOBO). The PCR reaction conditions were 95 °C for 5 min, 38 cycles at 94 °C for 15 s and 60 °C for 60 s. The internal control and the negative control were amplified using tubulin gene in soybean (GenBank: AY907703.1) and cDNA template was replaced with ddH_2_O, respectively. The normalized expression as fold changes for each line was calculated as ^△△*C*T^ = (C_T, Target_ − C_T, Tubulin_)_genotype_ − (C_T, Target_ − C_T, Tubulin_)_calibrator_.

### 4.4. Leaf Anatomical Structure by Transmission Electron Microscope

Preparation of electron microscope sample was conducted as previously described [38]. After low-P treatment for 14 days, leaf segments without veins (5–8/treatment) were dissected from randomly-selected seedlings and then fixed in 2.5% glutaraldehyde (*v/v*) overnight. Later, the samples were washed three times with the 0.1 M PBS solution (sodium phosphate buffer, pH 7.4). Then, every 15–20 min, the samples were dehydrated in ethanol with different concentrations (50–100%) and finally washed for 20 min by absolute acetone. Then, the samples were infiltrated and embedded in Spurr’s resin overnight. Heating at 70 °C for 9 h, ultrathin sections (70 nm, Leica EM UC6, Mannheim, Germany) of specimens were prepared and mounted on copper grids for viewing by a transmission electron microscope (JEOLTEM-1230EX) at an accelerating voltage of 40–120.0  kV.

### 4.5. Protein Extraction

Total protein was isolated from fully-developed soybean leaves. Leaves (500 mg) were pulverized into powder using liquid nitrogen. Then, 20 mM DTT in acetone and pre-cooled 10% (*w/v*) TCA were used to incubate at −20 °C for 1 h. The precipitated proteins were re-suspended and washed using ice-cold acetone containing 20 mM DTT until the supernatant was colorless. The protein pellet was dried under vacuum, suspended in buffer containing 2 M thiourea, 4% (*w/v*) 3-[(3-cholamidopropyl)dimethylammonio]-1-propane sulfonate (CHAPS), 7 M urea, cocktail protease inhibitor, and 0.2% (*v/v*) carrier ampholyte (pH 4.0–7.0). The insoluble tissue was removed by centrifugation at 15,000 *g* for 15 min (BECKMAN L-80XP, 90Ti, 90,000 rpm = 416,000 g). The supernatant was stored at −80 °C. The protocol reference [39] and lightly changed. Protein concentration was determined with bovine serum albumin (BSA) as a standard as previously reported [40].

### 4.6. Two-Dimensional Isoelectric Focusing (2D-IEF)/SDS-PAGE

2-DE was performed using pH 3–10, 24 cm immobilized pH gradient (IPG) strips (Bio-Rad, Hercules, CA, USA) and were 50v rehydrated at 20 °C for 15 h with 0.7 mg proteins and 300 μL liquid rehydration buffer (7 M urea, 4% (*w/v*) CHAPS, 0.2% (*v/v*) carrier ampholyte, 2 M thiourea, 1% (*w/v*) DTT) in every sample. Focusing was carried out in a Protein i12 IEF Cell (Bio-Rad, USA). The voltage procedure was as follows: (1) grade voltage increased to 50 V for 14 h; (2) grade voltage increased to 250 V for 30 min; (3) grade voltage increased to 1000 V for 1 h; (4) step voltage to 10,000 V for 5 h; (5) step voltage increased to 10,000 V and the focus increased to 60 kV h, and (6) grade voltage increased to 500 V for 2 h. Then, strips were equilibrated for 2 ×10 min in 6 M urea, 5% (*w/v*) SDS-PAGE and 30% (*v/v*) glycerol for the first equilibration step. For the second equilibration step, 2.5% iodoacetamide was used and then transferred onto a 12% polyacrylamide gel [41]. For each sample, gels were run in triplicate. Electrophoresis was performed in PROTEIN Plus Dodeca Cell (Bio-Rad, USA) according to the manufacturer’s recommendations. Protein molecular weight markers (Takara, Kusatsu, Japan) were used in the gel via a small piece of filter paper for calibration. The gels were stained with Coomassie brilliant blue red (CBB R-350) and decolorized with 5% acetic acid [42].

### 4.7. Image and Statistical Analysis

Image acquisition was performed using UMAX PowerLook 2100 XL (Flatbed Scanner, UMAX Technologies Inc., Dallas, TX, USA). For all samples, three replicate gels were analyzed, and the greatest amount of protein spots for the third image was selected as the representative reference gel. Image analysis was performed with PDQuest software (v730, Bio-Rad, Hercules, CA, USA). In order to compensate subtle differences in sample loading, destaining, and gel staining, the spot volume was normalized as the relative volume. Then high-level match sets were created to compare the results of different experiments and annotate differential spots. The Student*’*s *t*-test was used to test the statistical significance of the quantitative data at a 95% confidence level. A 1.5-fold or more change in protein concentration between the two P levels were considered to be differentially accumulated.

### 4.8. Peptide Mass Fingerprinting (PMF) Analysis

After proteolysis, the spots were tested using MALDI-TOF MS (matrix-assisted laser desorption/ionization time of flight mass spectrometry) and identified by peptide mass fingerprinting (PMF), as previously described [43]. The list of peptide masses was transferred into the peptide mass fingerprint as a data file [44], and the simulated proteolysis and fragmentation of known proteins in the non-redundant Soybase database (http://www.soybase.org/) [45]. The results were retrieved and analyzed according to the score, E-value, mass, coverage rate, number of matching peptides, experimentally-calculated P and other factors [29].

### 4.9. Statistical Analysis

Statistical analysis was performed using SAS 9.2 (SAS Institute Inc., Cary, NC, USA). Means of the values for the three replicates were compared using the *t*-test or variance analysis (ANOVA) depending on the number of treatments being compared.

## 5. Conclusions

Our results showed that low-P stress can inhibit photosynthesis capacity and growth of soybean plants by inhibiting ATP synthesis and carbon assimilation, damaging the structure of PSII, and impairing the leaf anatomical structure. Our results shed light on the mechanisms involved in the response to low-P stress in soybean and indicated the involvement of P efficiency and supply in photosynthesis at the levels of physiology, phenotype, gene, and protein relative abundance. Future studies will be conducted to clarify the molecular mechanism of how P ameliorates the effects on soybean photosynthesis.

## Figures and Tables

**Table 1:** Phenotypic variation of photosynthetic- and P efficiency-related traits in soybean.

Traits Name (Units)	Traits Abbreviation	Means ± SD	Means ± SD
		B20 + P	B20 − P
**Growth-related traits**			
Shoot dry weight (g)	SDW	0.424 ± 0.022 aA	0.352 ± 0.047 bB
Root dry weight (g)	RDW	0.073 ± 0.00 bB	0.096 ± 0.008 aA
Total dry weight (g)	TDW	0.497 ± 0.019 aA	0.448 ± 0.053 bA
Root: shoot ratio	R/S	0.172 ± 0.0174 bB	0.274 ± 0.027 aA
Acid phosphatase activity	APA	1.33 ± 0.12 aA	3.24 ± 0.40 bB
ATP synthase (μmolPi·mgprot^−1^·h^−1^)	ATPase	18.48 ± 0.92 aA	16.27 ± 0.94 aA
Ribulose-1,5-bisphosphate carboxylase	RuBPase	37.31 ± 1.02 aA	31.41 ± 0.95 bB
**P efficiency-related traits**			
Phosphorus concentration (mmol·L^−1^)	PC	23.47 ± 0.99 aA	7.19 ± 0.69 bB
Phosphorus absorption efficiency	PUP	11.67 ± 0.88 aA	3.24 ± 0.64 bB
Phosphorus use efficiency	PUE	0.04 ± 0.01 bB	0.14 ± 0.01 aA
**Photosynthesis-related traits**			
Chlorophyll content (mg·g^−1^)	CC	32.2 ± 0.72 aA	28.9 ± 1.65 aA
Net photosynthetic rate (μmol·m^2^·s^−1^)	Pn	17.60 ± 1.05 aA	12.37 ± 0.50 bB
Stomatal conductance (mmol·m^−2^·s^−1^)	Gs	210 ± 8.19 aA	240.33 ± 5.51 bB
Transpiration rate (g· m^−2^·h^−1^)	Tr	4.44 ± 0.35 aA	2.97 ± 0.46 bB
Intercellular CO_2_ concentration	Ci	0.25 ± 0.04 aA	0.19 ± 0.01 bB
Maximum quantum efficiency of PSII	Fv/Fm	0.82 ± 0.01 aA	0.72 ± 0.01 bB
Quantum efficiency of PSII	ΦPSII	0.54 ± 0.02 aA	0.50 ± 0.01 bA
Photochemical quenching	qP	0.72 ± 0.02 aA	0.62 ± 0.03 bB
Non-photochemical quenching	NPQ	0.38 ± 0.02 aA	0.46 ± 0.02 bB

SD denotes standard deviation; B20 optimum P/−P denote soybean varieties B20 were grown at optimum/low-P conditions; ANOVA was used to compare significance and the lowercase/capital letter denote significantly different at *p* < 0.05/0.01 (LSR test). The same lowercase/capital letter denote that the difference is not significant.

**Table 2:** Relative transcript levels by transcriptome and validation by *q*PCR of photosynthetic-related genes under different P levels.

Genes	Fold Change (−P/+P)		Expression	Annotation
	*q*PCR	Transcriptome		
*Glyma.01G095900*	1.32	2.43	down	Photosystem II PsbZ protein
*Glyma.03G065000*	3.33	10.07	down	Photosystem II PsbH protein (PsbH)
*Glyma.03G068100*	5.89	2.07	down	Ribulose-1,5-bisphosphate carboxylase
*Glyma.05G022900*	2.79	8.75	down	Photosystem I subunit III (PsaF)
*Glyma.06G277500*	1.79	2.16	down	Phosphoenolpyruvate carboxylase
*Glyma.08G201200*	1.29	4.50	up	NADP^+^-dependent malic enzyme
*Glyma.12G096200*	1.56	6.80	down	ATP synthesis coupled proton transport
*Glyma.13G149800*	1.19	2.20	up	Pyruvate kinase activity
*Glyma.15G188400*	1.51	3.71	down	Photosystem II (PsbN)
*Glyma.15G248600*	1.72	1.42	down	Photosystem II PsbK protein
*Glyma.17G020600*	2.04	2.03	down	Pyruvate orthophosphate dikinase
*Glyma.18G036400*	1.63	2.51	down	Rubisco activase
*Glyma.19G190100*	1.27	2.14	up	Pyruvate kinase, barrel domain
*Glyma.20G122500*	1.33	2.15	down	Fructose-biphosphate aldolase

**Table 3:** List of 17 photosynthetic related proteins with significantly different abundance in the leaves of soybean in response to low-P stress.

Spot No.	Gene ID	Matched Protein Name	Pattern	*pI*	*Mw*	Coverage Rate (%)	Protein Score	FC (−P/+P)
S1	*Glyma.11G108500*	Chloroplast movement	Up	4.52	95.2	33.45	73.1	1.57
S2	*Glyma.06G277500*	Phosphoenolpyruvate carboxylase	Up	5.33	75.1	30.60	67.49	2.05
S3	*Glyma.19G224200*	Phytochrome A	Up	6.69	94.6	28.56	68.94	2.03
S4	*Glyma.16G173100*	Pyruvate kinase	Up	6.54	62.2	38	73.32	6.65
S5	*Glyma.19G186000*	Triosephosphate isomerase (TPI)	Down	6.33	33.2	45.60	65.4	0.36
S6	*Glyma.10G197700*	Malate dehydrogenase activity	Up	6.63	48.8	19.90	69.9	8.60
S7	*Glyma.13G078600*	ATP synthase alpha/beta family	Down	6.62	24.4	19.68	70.3	0.52
S8	*Glyma.18G036400*	Rubisco activase	Down	8.01	45.5	7.35	66.26	/
S9	*Glyma.08G142700*	Iron-sulfur cluster assembly protein	Down	7.99	18.1	11.50	71.65	0.50
S10	*Glyma.20G090000*	Phytochrome A	Down	6.35	15.2	6.30	69.58	0.23
S11	*Glyma.06G091500*	Phosphoenolpyruvate carboxykinase	Up	7.55	70.9	8.59	63.2	/
S12	*Glyma.05G025300*	Ribulose-phosphate 3-epimerase	Up	7.89	29.6	21.80	64.75	/
S13	*Glyma.05G022900*	Photosystem I subunit III	Up	5.01	28.6	4.90	61.49	/
S14	*Glyma.15G266300*	Myosin ATPase	Up	8.69	17.6	24.10	73.6	/
S15	*Glyma.15G018000*	Electron carrier activity	Up	8.58	22.3	35.20	72.63	/
S16	*Glyma.07G185400*	Malate dehydrogenase	Down	5.82	37.0	28.40	77.26	0.31
S17	*Glyma.13G089200*	Cryptochrome 1	Down	6.32	77.0	2.90	62.19	/

FC: fold change (the relative protein spot volume of −P treatment to the relative protein spot volume of optimum P treatment). The proteins were identified by peptide mass fingerprinting (PMF) and BLASTed in Phytozome and NCBI databases. *pI,* polydispersity index; *Mw*, molecular weight.

## Supplementary Materials

Supplementary materials can be found at http://www.mdpi.com/1422-0067/19/6/1688/s1.

## Abbreviations

APA	Acid phosphatase activity
ATPase	ATP synthase
CC	Chlorophyll content
Ci	intercellular CO_2_ concentration
Fv/Fm	Maximum quantum efficiency of PSII
Gs	Stomatal conductance
NPQ	Non-photochemical quenching
PAE	Phosphorus absorption efficiency
PC	Phosphorus concentration
Pn	Net photosynthetic rate
PUE	Phosphorus use efficiency
qP	Photochemical quenching
RDW	Root dry weight
RuBPase	Ribulose-1,5-bisphosphate carboxylase
R/S	Root: shoot ratio
TDW	Total dry weight
Tr	Transpiration rate
SDW	Shoot dry weight
ΦPSII	Quantum efficiency of PSII
