# Supplementary figures and images for: Physiological and Proteomics Analyses Reveal Low-Phosphorus Stress Affected the Regulation of Photosynthesis in Soybean
Source: Int J Mol Sci. 2018 Jun 6;19(6):1688. doi: 10.3390/ijms19061688 (PMC6032344; doi:10.3390/ijms19061688)

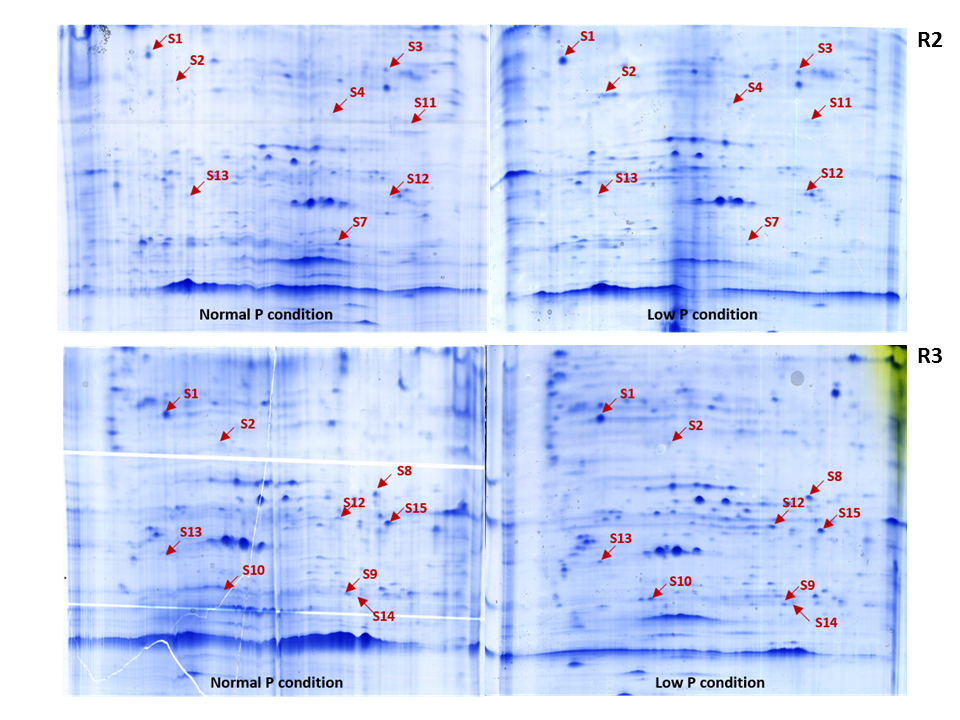

Supplement: Supplementary file 1 [file ijms-19-01688-s001.zip › Figure S1.tif]

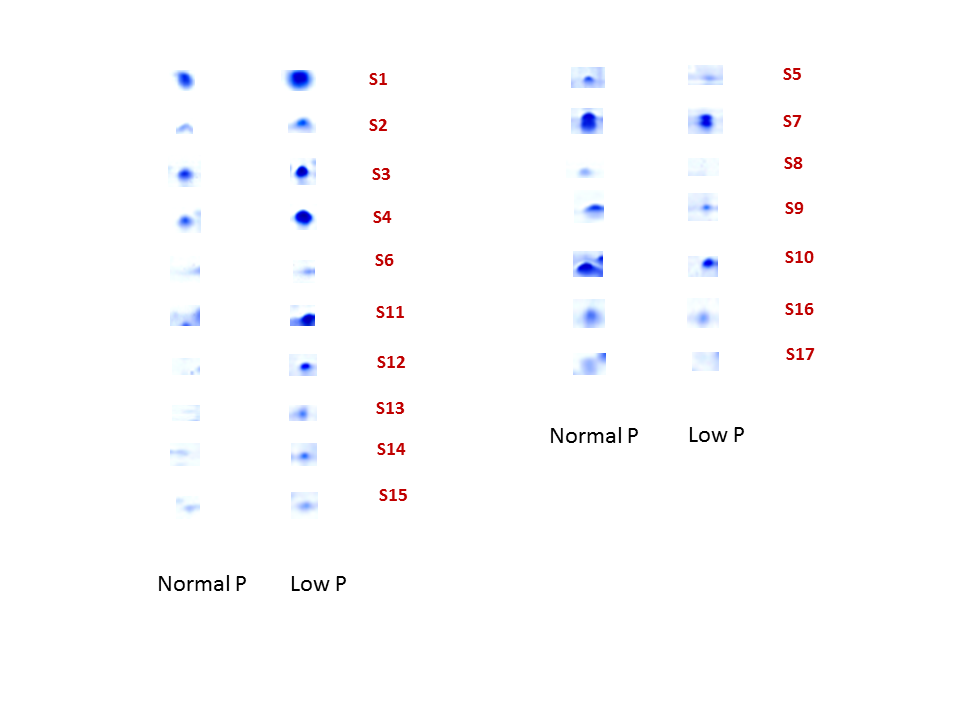

Supplement: Supplementary file 1 [file ijms-19-01688-s001.zip › Figure S2.tif]
